# Effects of a Residential Multimodal Psychological Treatment in an Addicted Population, at 6 and 12 Months: Differences Between Men and Women

**DOI:** 10.3389/fpsyt.2022.862858

**Published:** 2022-06-16

**Authors:** Asunción Santos-de-Pascual, Luis Miguel López-Cano, Mavi Alcántara-López, Antonia Martínez-Pérez, Maravillas Castro-Sáez, Visitación Fernández-Fernández, Concepción López-Soler

**Affiliations:** ^1^Solidarity and Reinsertion Foundation, Man Project of Murcia, Murcia, Spain; ^2^Faculty of Psychology, University of Murcia, Murcia, Spain

**Keywords:** addiction, substance-related disorders, treatment, retention, gender

## Abstract

The aim of this study is to explore the effects of a residential multimodal treatment intervention for an addict population. We gathered participants from the “Programa Base” (*n* = 166) of the Solidarity and Reinsertion Foundation of Murcia, and assessed the various problematic areas with the EuropASI at baseline level, 6 months and 12 months of treatment. We found improved outcomes in every area except for Legal Status. In addition, we found differences between male and female participants in their baseline evaluation, as well as between completers and non-completers. In conclusion, this data shows us some changes which occurred in individuals with problematic drug use during treatment, going further into the complex social reality which causes great suffering and damage to people and their families.

## Introduction

Drug use continues to be one of the most persistent threats to health in Spain and Europe, directly and indirectly affecting the well-being of millions of people in our country (1). Understanding of the phenomenon of addictions has increased in recent years, thanks to the continuous effort of professionals and researchers to elucidate the most relevant treatment factors as well as the current commitment to investing in effective treatment approaches (1–3).

There have been several publications over the last 30 years on the evidence of the effectiveness of Therapeutic Communities (TCs) for addiction treatment with publications on this subject in systematic reviews and meta-analyzes (4–11).

The review by Malivert et al. (6), aimed to assess the effectiveness of TC treatment on abstinence and determine its predictive factors. Twelve studies were selected in which 3,271 patients from 61 TCs participated. All studies showed a reduction in substance use during treatment and following discharge. Treatment completion was the best predictor of abstinence at follow-up, though long-term benefits were uncertain. There were important complications when comparing data due to the large diversity between treatment modalities evaluated, duration, and characteristics of the population who attended.

Various studies have found a strong link between treatment duration in TCs and completion, with greater recovery than when treatment is abandoned prematurely (12–17). Therefore, treatment abandonment is of great concern in all addiction treatment modalities (18), and is often most common in the first months (11). This is particularly relevant when we consider that TCs appear less effective than other forms of intervention regarding treatment adherence (18), and that results improve every 3 months that a person remains (7, 19, 20).

In addition to treatment, type and duration influence outcome and results. Recent research indicates that addiction severity, gender, attachment, comorbidity with personality disorders, therapeutic alliance, relationships within TCs, and social and occupational integration are highly relevant variables when predicting treatment results and adherence in the first months (21–34).

The vast majority of studies in systematic reviews and meta-analytic studies were carried out on the American and Australian population, although 2 studies on the Spanish population are also mentioned. In the work by Fernández-Montalvo et al. (35), a long-term follow-up was performed (a mean of 6 years following treatment) of a TC treatment for addictions. A comparison was made between those who completed and who abandoned treatment. The sample comprised 155 subjects (113 who completed and 42 who dropped out). The latter showed a higher and earlier rate of both relapses (83.3 vs. 32.7%) and new treatments for their addiction than the completion group (66.7 vs. 23%). The program was also effective in reducing illegal behavior and improving health status. In the second study by Fernández-Hermida et al. (36), the authors found significant reductions in the use of alcohol and illegal drugs, in illegal behavior, and a large percentage of those assessed, achieved and maintained stable employment at the 3-year follow-up. The main differences were found between the completion group and those who abandoned it with the latter suffering relapses in a much shorter period than the rest.

As well as studies included in reviews, there are relevant works by López-Goñi et al. (37), Pérez del Río (38) and Valero-Agüayo (39), for treatments similar to those analyzed in previous meta-analytical studies (35, 36). López-Goñi et al. (37), described the pre-post treatment evolution of a sample of 112 patients observed in two Spanish TCs. Sample evaluation was with the Addiction Severity Index (ASI), at start and finish of treatment. Sixty nine point seven percent of the sample completed treatment and 30.3% abandoned it. Results showed a statistically significant improvement in eight of the nine areas evaluated. Only in Physical Health were there no significant changes. The authors highlight that this is possibly due to the high rate of chronic physical illnesses presented by those evaluated. Pérez del Río (38) found that people with better prognosis and who complete 6 months of treatment tend to respond to less unstructured profiles at the social and relational level, have experienced longer abstinence periods, and had significantly lower drug use. On the other hand, Valero-Agüayo et al. (39) observed that the presence of polydrug use, emotional and physical abuse, and numerous family conflicts were factors closely related to abandonment, thus suggesting treatment models that take these variables into account.

Our main aim is to analyze the efficacy of a multicomponent treatment protocol in people with severe substance use disorder, as well as to observe the influence of time in treatment in critical areas (medical health, employment problems, alcohol problems and illegal drugs, social and family relationships, and psychopathological state). Our main hypothesis is that the longer a person remains in treatment, the more pronounced the decrease in addiction severity and other associated problems.

Our second aim is to analyze the relationship between women and men, type of completion, and number of treatments with addiction severity and other associated problems.

## Methods

### Participants

The study simple was drawn from the 322 cases attended to between January 2017 and January 2019 in the base program of the Solidarity and Reintegration Foundation of the Region of Murcia. Inclusion criteria were: (a) have substance use problems, (b) participate voluntarily. An exclusion criterion was that participants had not been in treatment for substance use disorder in the previous 6 months.

On applying these criteria, the selected sample was 166 participants (51.55% of the initial population). The mean age of people in treatment was 40.17 years. 22.3% were women. The main substance of consumption in the sample was cocaine (29.5%), followed by joint consumption of alcohol and other drugs (22.9%), and alcohol alone (16.3%). 38.6% reported having received some prior treatment. The mean age and standard deviation at the start of consumption was 26.37 (SD = 21.36) and 21 years of habitual consumption (Table 1).

**Table 1:** Sociodemographic characteristics of sample.

**Variable**	***N* (%)**	***N* men (%)**	***N* women (%)**
Sex			
Men	129 (77.7)	-	-
Women	37 (23.3)	-	-
Civil status			
Married	26 (15.7)	23 (17.8)	3 (8.1)
Widowed	1 (0.6)	-	1 (2.7)
Divorced	33 (19.9)	20 (15.5)	13 (35.1)
Single	91 (54.8)	74 (57.4)	17 (45.9)
Education level			
Primary	28 (16.9)	22 (17.1)	6 (16.2)
Secondary	51 (30.7)	40 (31)	11 (29.7)
Higher	9 (5.4)	5 (3.9)	4 (10.8)
Legal status			
No legal problems	138 (83.13)	105 (81.39)	33(89.18)
With some legal problem	28 (16.9)	24 (18.8)	4 (10.8)
Employment type			
Full time	99 (59.6)	80 (62)	19 (51.4)
Part-time (stable)	15 (9)	8 (6.2)	7 (18.9)
Part-time (irregular, temporary)	11 (6.6)	10 (7.8)	1 (2.7)
Student	4 (2.4)	3 (2.3)	1 (2.7)
Retired/disability	6 (3.6)	4 (3.1)	2 (5.4)
Unemployed	8 (4.8)	6 (4.7)	2 (5.4)
Safe environment	5 (3)	5 (3.9)	-
Source of income			
Employment	36 (21.7)	27 (20.9)	9 (24.3)
Social security	12 (7.2)	10 (7.8)	2 (5.4)
Social aid	5 (3)	4 (3.1)	1 (2.7)
Penson or social security	28 (16.9)	21 (16.3)	7 (18.9)
Partners, family or friends	46 (27.7)	35 (27.1)	11 (29.7)
Ilegal	1 (0.6)	1 (0.8)	-
Other sources	8 (4.8)	5 (3.9)	3 (8.1)

The absence of substance use was assessed by self-report and by random urine sample tests throughout the therapeutic process.

### Instruments

Sociodemographic variables: information on user status was gathered through a semi-structured registry interview developed for this work. Sociodemographic data were gathered (gender, age, educational level, employment, legal records) and clinically relevant variables (substances used, family history of use, type of main substance used, years of use, number of previous treatments).

The EuropASI: is the European Version of the Addiction Severity Index, ASI (40) and the Spanish version was used for this study (41), which presents high internal consistency (α = 0.70). It is a hetero-applied, semi-structured, clinical interview totaling 159 items, widely used for evaluation and diagnosis of patients upon admission to treatment programs, which explores six areas of special relevance to addiction problems: physical health, employment and resources, alcohol and/or drug consumption, legal status, family and social relationships and psychopathological state. As well as different items for each of these areas, the instrument provides a severity index ranging from 0 (no problem) to 9 (extreme problem), with highest scores indicating the need for treatment.

### Treatment Program

The Solidarity and Reintegration Foundation is a non-governmental organization providing treatment and rehabilitation programs for those with substance use problems and behavioral addictions. The program evaluated in this study, the Base Program (BP), is a semi-residential treatment program whose intervention is based on the biopsychosocial model, and whose aims are: (a) achievement and maintenance of abstinence by users, (b) normalization and social reintegration of user regarding work and family, and (c) The adoption of a lifestyle promoting personal autonomy.

The treatment program comprises two phases. The reception phase lasts between 4 and 6 months approximately and is characterized by intensive group work of a cognitive-behavioral type oriented toward maintaining abstinence and adherence to treatment. Users were divided according to therapeutic criteria, providing access to residential treatments to more serious cases, people who could not stay on their own or the family home, or did not have a person of reference to help during the process.

The therapeutic community (TC) phase is conducted in a residential way, providing a micro-society where residents and a team of professionals, as a facilitating instrument, assume different roles and are governed by clear and specific rules, designed to promote the personal evolution of residents. Intervention is focused on analysis of various factors involved in relapse prevention and lasts between 6 and 8 months.

The treatment program consists of various components mainly developed through group dynamics, in daily group and individual support sessions when required, throughout the process. Components are: Coexistence program; Cognitive restructuring; Social skills training; Self-regulation/self-control; Recognition and emotional expression; Reconstitution of personal identity; Restructuring of securities; Relapse prevention; Family therapy; Contingency management.

### Data Analysis

Descriptive statistics were first obtained from sociodemographic data and the clinical variables of the sample. To analyze the relationship between sociodemographic and clinical characteristics, independent mean comparisons were performed using the Mann-Whitney U test. To assess results of the program, analysis of comparison of means related by the Wilcoxon signed-rank test was conducted. To calculate the effect size of the non-parametric tests, r (r = Z / √N) was used in accordance with the procedure described by Rosenthal (1994) when assumptions to calculate Cohen's cannot be fulfilled.

All analyzes and data treatments were performed using the statistical package SPSS 21.0.

## Results

Of the 166 people evaluated at start of treatment, 42.8% abandoned treatment before completion, while 50% remained to the end (see Table 2). In the longitudinal analysis of the different moments of evaluation of treatment (baseline, 6 months and 12 months), relevant data was found on the effect of treatment on severity of patients' problems. In comparison between baseline and 6 months, significant differences were found in Physical Health indices (*Z* = 5.290, *p* < 0.01, *r* = 0.526), Employment Problems (*Z* = 4.033, *p* < 0.01, *r* = 0.401), Alcohol Use (*Z* = 5.778, *p* < 0.01, *r* = 0.575), Drug Use (*Z* = 7.029, *p* < 0.01, *r* = 0.699), Social / Family Relations (*Z* = 4.873, *p* < 0.01, *r* = 0.485), and Psychopathological State (*Z* = 5.853, *p* < 0.01, *r* = 0.582) (see Table 3), 42.8% abandoned treatment before completion.

**Table 2:** Substance use and clinically relevant variables.

**Variable**	***n***	**%**
Retention		
Drop out deliberate	74	44.6
Completers	83	50
Expulsion	6	3.6
Referral	2	1.2
Other reasons	1	0.6
Main consumption substance		
Alcohol	27	16.3
Heroin	10	6
Other opioids/pain relievers	2	1.2
Benzodiazepines and other	2	1.2
Cocaine	49	29.5
Cannabis	8	4.8
Other	2	1.2
More than one substance daily	3	1.8
Alcohol and drugs	38	22.9
Polytoxic addict	4	2.4
Previous Treatments	64	38.6
Associated psychological problems		
Depressed mood	13	7.8
Hostile mood	3	1.8
Anxiety or nervousness	8	4.8
Thought disturbance, paranoid ideation	154	92.8
Attention or memory disturbance	13	7.8
Suicidal ideation	2	1.2

**Table 3:** Differences between baseline evaluations and 6 months.

	**Area**	**Mean (SD)**	**Z (p)**	***r***	***n***
Baseline	Physical health	3.73 (2.848)	5.290 (0.001^**^)	0.526	101
6 months		2.87 (2.509)			
Baseline	Employment problems	5.71 (3.043)	4.033 (0.001^**^)	0.401	101
6 months		4.54 (2.917)			
Baseline	Alcohol use	6.08 (2.989)	5.778 (0.001^**^)	0.575	101
6 months		4.47 (2.352)			
Baseline	Drug use	7.11 (2.894)	7.029 (0.001^**^)	0.699	101
6 months		5.01 (2.555)			
Baseline	Legal status	2.14 (3.158)	0.135 (0.893)	0.013	101
6 months		2.10 (3.008)			
Baseline	Social/family relations	6.81 (2.114)	4.873 (0.001^**^)	0.485	101
6 months		5.35 (1.802)			
Baseline	Psychopathological state	6.8 (2.413)	5.853 (0.001^**^)	0.582	101
6 months		5.19 (2.279)			
6 months	Physical health	2.87 (2.509)	3,163 (0.002^**^*)	0.356	79

** *p < 0.01. Bold values are to highlight significant results*.

Similar decreases were found in severity indices on comparing evaluation at 6 months with that of 12 months, in Physical Health indices (*Z* = 3,163, *p* < 0.01, *r* = 0.356), Employment Problems (*Z* = 5,028, *p* < 0.01, *r* = 0.566), Alcohol Use (*Z* = 5.207, *p* < 0.01, *r* = 0.586), Drug Use (*Z* = 6.797, *p* < 0.01, *r* = 0.511), Social / Family Relations (*Z* = 4.455, *p* < 0.01, *r* = 0.501), and Psychopathological State (*Z* = 4.988, *p* < 0.01, *r* = 0.561) (see Table 4).

**Table 4:** Differences between 6-month and 12-month evaluations.

	**Area**	**Mean (SD)**	**Z (p)**	***r***	***n***
6 months	Physical health	2.87 (2.509)	3,163 (0.002^***^)	0.356	79
12 months		2.58 (2.246)			
6 months	Employment problems	4.54 (2.917)	5,028 (0.001^**^)	0.566	79
12 months		3.41 (2.524)			
6 months	Alcohol use	4,47 (2.352)	5,207 (0.001^**^)	0.586	79
12 months		3.27 (2.191)			
6 months	Drug use	5.01 (2.555)	4,538 (0.001^**^)	0.511	79
12 months		3.56 (2.515)			
6 months	Legal status	2.10 (3.008)	1,245 (0.213)	0.140	79
12 months		1.98 (2.603)			
6 months	Social/family relations	5.35 (1.802)	4,455 (0.001^**^)	0.501	79
12 months		4.41 (2.190)			
6 months	Psychopathological state	5.19 (2.279)	4,988 (0.001^**^)	0.561	79
12 months		4.40 (2.225)			
6 months	Physical health	2.87 (2.509)	5.091 (0.001^**^)	0.566	81
12 months		2.58 (2.246)			

** *p < 0.01. Bold values are to highlight significant results*.

As for treatment effects when comparing the initial and 12-month evaluations, significant differences were found in Physical Health indices (*Z* = 5.091, *p* < 0.01, *r* = 0.566), Employment Problems (*Z* = 5.369, *p* < 0.01, *r* = 0.597), Alcohol Use (*Z* = 6.735, *p* < 0.01, *r* = 0.748), Drug Use (*Z* = 6.797, *p* < 0.01, *r* = 0.748), Social/Family Relations (*Z* = 4.873, *p* < 0.01, *r* = 0.631), and Psychopathological State (*Z* = 4.988, *p* < 0.01, *r* = 0.702) (see Table 5).

**Table 5:** Differences between baseline and 12-month evaluations.

	**Area**	**Mean (SD)**	**Z (p)**	***r***	***n***
Baseline	Medical status	2.87 (2.509)	5.091 (0.001^**^)	0.566	81
12 months		2.58 (2.246)			
Baseline	Employment problems	4.54 (2.917)	5.369 (0.001^**^)	0.597	81
12 months		3.41 (2.524)			
Baseline	Alcohol use	4,47 (2.352)	6.735 (0.001^**^)	0.748	81
12 months		3.27 (2.191)			
Baseline	Drug use	5.01 (2.555)	6.797 (0.001^**^)	0.748	81
12 months		3.56 (2.515)			
Baseline	Legal status	2.10 (3.008)	0.698 (0.485)	0.077	81
12 months		1.98 (2.603)			
Baseline	Social/family relations	5.35 (1.802)	5.677 (0.001^**^)	0.631	81
12 months		4.41 (2.190)			
Baseline	Psychopathological state	5.19 (2.279)	6.315 (0.001^**^)	0.702	81
12 months		4.40 (2.225)			

** *p < 0.01. Bold values are to highlight significant results*.

Significant differences were found between men and women in several of the EuropASI severity indices (see Table 6). A greater severity of problems was observed among women in the Physical Health index (UMW = 1667.5; *p* < 0.01; *r* = 0.29), Social/Family Relations (UMW = 1762.0; *p* < 0.05; *r* = 0.19), and Psychopathological State (UMW = 1756.0, *p* < 0.012, *r* = 0.19), and a greater severity in the group of men in Drug Use (UMW = 1876.0; *p* < 0.05; *r* = 0.16), and Legal Status (UMW = 1903.0; *p* < 0.05; *r* = 0.16).

**Table 6:** Differences by sex.

	**Area**	**Mean (SD)**	**Mann Whitney U Test (p)**	***r***	***n* (%)**
Men	Medical status	3.41 (2.732)	1667.5 (0.005^**^)	0.29	129(77.7%)
Women		4.86 (2.992)			37 (22.3%)
Men	Drug use	7.44 (2.546)	1876.0 (0.035^*^)	0.16	129(77.7%)
Women		5.95 (3.681)			37 (22.3%)
Men	Legal status	2.36 (3.243)	1903.0 (0.039^*^)	0.16	129(77.7%)
Women		1.35 (2.741)			37 (22.3%)
Men	Social/family relations	6.62 (2.137)	1762.0 (0.014^*^)	0.19	129(77.7%)
Women		7.49 (1.909)			37 (22.3%)
Men	Psychopathological state	6.54 (2.559)	1756.0 (0.012^*^)	0.19	129(77.7%)
Women		7.70 (1.525)			37 (22.3%)

* *p < 0.05*,

** *p < 0.01. Bold values are to highlight significant results*.

On analyzing differences found at the start of treatment of those who completed and who abandoned treatment, differences were only seen in Social / Family Relations (UMW = 2349.5; *p* < 0.05; *r* = 0.18) (see Table 7). On comparing the group who had previously received treatment with those starting treatment for the first time, significant differences were found in Drug Use at 6 months of treatment (UMW = 934.50; *p* < 0.05; *r* = 0.21) and at 12 months (UMW = 529.00; *p* < 0.05; *r* = 0.28), but notat the beginning.

**Table 7:** Differences by number of previous treatments, and type of discharge.

	**Area**	**Mean (SD)**	**Mann Whitney U Test (*p*)**	***r***	***n* (%)**
No prior **T**_**x**_	Drug use (Start)	6.95 (2.973)	3070.00 (0.493)	0.05	102 (61.5%)
Prior **T**_**x**_		7.36 (2.768)			64 (38.5%)
No prior **T**_**x**_	Drug use (6 months)	4.59 (2.520)	934.50 (0.034^*^)	0.21	59 (58.4%)
Prior **T**_**x**_		5.60 (2.519)			42 (41.6%)
No prior **T**_**x**_	Drug use (12 months)	2.98 (2.338)	529.00 (0.011^*^)	0.28	48 (59.3%)
Prior **T**_**x**_		4.39 (2.768)			33 (40.7%)
Voluntary discharge	Social relations/relatives	7.10 (2.185)	2349.5 (0.027^*^)	0.18	71 (46.1%)
Therapeutic discharge		4.86 (2.992)			83 (53.9%)

* *p < 0.05. Bold values are to highlight significant results*.

There are no significant differences between the Medication/No-Medication groups for any EuropASI indices at any evaluation time (Baseline, 6 months, 12 months), except for the index of legal problems at Baseline evaluation (UMW = 2456, *p* < 0.05, *r* = 0.17) and at 12 months (UMW = 438.5, *p* < 0.05, *r* = 0.29), and the index of psychological problems at 6 months (UMW = 709, *p* < 0.01, *r* = 0.29) (see Table 8).

**Table 8:** Differences by medication status.

	**Area**	**Means (SD)**	**Mann–Whitney *U* test (*p*)**	***r***	***n* (%)**
No_Med	Legal status (Start)	1.6 (2.685)	2,456 (0.037^*^)	0.17	71 (45.81%)
Med		2.51 (3.389)			84 (54.19%)
No_Med	Psychopathological state (6 m)	4.5 (2.325)	709 (0.006^**^)	0.29	48 (52.17%)
Med		5.84 (2.145)			44 (47.83%)
No_Med	Legal status (12 m)	2.28 (2.501)	438.5 (0.016^*^)	0.29	40 (55.56%)
Med		1.16 (2.245)			32 (44.44%)
No_Prob	Psychopathological state (Start)	6.21 (2.601)	1762.0 (0.014^*^)	0.16	92 (59.36%)
Prob_Con		7.12 (2.329)			63 (40.64%)
No_Prob	Legal status (12 m)	1.71 (2.44)	435 (0.013^*^)	0.29	39 (54.17%)
Prob_Con		1.81 (2.464)			33 (45.83%)

* *p < 0.05*,

** *p < 0.01*.

There are differences in the EuropASI index of psychological problems in the evaluation of Start (UMW = 2,372, *p* < 0.05, *r* = 0.16) between people who manifested problems with concentration in the previous month and those who did not, and in problems 12 months after treatment (UMW = 435, *p* < 0.05, *r* = 0.29).

No significant differences were found for any of the indices between people who experienced severe depression and those who did not. Nor for those who manifested severe anxiety or who experienced violent behavior.

The difference between pretreatment (baseline) and posttreatment (12 months), in substance use was 3.4 (SD: 2.83), effect size is equal to 1.2 (d), and power is 1 for the final sample.

## Discussion

The evaluated sample is similar to those found in other articles in the field of addictions (4, 6–8, 18, 42), commonly finding a high percentage of men, cocaine and alcohol users, and polydrug users. The retention ratio in this study is in line with other research (6, 8, 18).

It is worth noting the high percentage of users presenting psychopathological problems, 92.8% symptoms of mental pathology, far from that found in other studies, which report prevalences of around 50% (24, 43–47). This difference might be due to the ASI not being a diagnostic tool and that its psychopathological area is limited to exploring and providing information on possible problems, i.e., it is a screening tool, although it may also be explained by the fact that those who choose this resource (Foundation for Reintegration...) have a longer and more complex history of consumption. Thus, these data must be taken with caution and this area should be explored in future studies with standardized diagnostic tests that can more reliably estimate psychological pathology.

The most striking treatment results have always been found when comparing severity of problems of profiles at start of treatment with results after 12 months. These data correspond to those in other studies on the importance of time spent in treatment (12–17). In this study, the severity of medical problems and those related to illegal substances (EuropASI Medical and EuropASI Drugs) reduced considerably in the first 6 months of treatment. Contrarily, in severity of family/social and employment problems, greater treatment effects are observed in the second period (6–12 months). This more pronounced decrease in the last stage might be due to the fact that vocational training and/or employability workshops do not begin until severity of alcohol and drug use has decreased, as occurs within the area of relationship problems between users and their family members (6, 8, 18). These data, added to the fact that the only difference we found between those completing treatment and those not, was greater severity of family/social problems, supporting the need for treatment modalities that include interventions in this area. These must be developed from the very start as delay until later intervention stages can cause intense emotional burden in users that can lead to premature abandonment of treatment. This is relevant if we consider that the person's level of social and family integration plays a key role in whether they remain in treatment (6, 8, 32, 34, 48, 49).

No variations were found regarding legal problems throughout treatment. We understand this is caused by the low severity of legal problems presented by the collected sample, since other treatment modalities in TCs did find a marked reduction in these problems among prison populations that present high levels of this type PODEMOS QUITAR CONCHA? (8, 50–52).

There are considerable differences between men and women in the entry profile as regards severity of medical, family, psychological, legal problems, and dependence on illegal substances. As the percentage of women in the sample was low, data should be viewed with caution.

In this study, it was found that patients with a history of previous treatments obtain worse results at 6 months and 12 months in the EuropASI Drug Use Index, corresponding to data that indicate better results with fewer treatment episodes (53–56). Some studies mention the difficulty of profiles with several previous failed treatment schedules (54, 55, 57, 58) which may be due to greater severity at start of treatment. Darke et al. (59), found that users who had stayed longer in the same treatment itinerary obtained better results, while those presenting a longer history of previous treatments, and with a similar or even longer time in treatment often achieved much worse treatment results (26, 29, 54, 55, 57, 58).

As for the study's limitations, a control group could not be included in the research due to the desire to provide users with the best possible treatment without delay, which is a methodological weakness, as well as the absence of longer follow-up. Nevertheless, it provides relevant data on two treatment stages which can help improve the applied protocols.

## Conclusions

The main conclusions are as follows: (a) longer treatment time brings better therapeutic results; (b) severity of treatment areas related to substances and physical health greatly improve in the first months of treatment, while social/ family and employment problems require longer for improvement to be effective; (c) Patient gender influences severity of consumption problems, legal situation, physical health, and the social/family relationships presented and these must be considered when designing interventions; (d) Social-family problems influence retention of treatment and these problems must be addressed from the start to try and prevent premature abandonment of treatment; (e) Those with a longer history of treatment present additional difficulties and these must be determined to avoid early abandonment.

The main aim of this research was to assess the efficacy of the multimodal cognitive-behavioral treatment protocol applied at the Solidarity & Reinsertion Foundation of Murcia (Murcia), and is a first step toward knowing the effects throughout treatment for 1 year in people with consumption problems, delving into this social reality that brings so much suffering and deterioration to people and their families. Information on follow-up at 18 and 24 months is being gathered in this sample, owing to the importance of maintaining improvement achieved by treatment and of obtaining more information on differences between men and women.

## Data Availability Statement

The original contributions presented in the study are included in the article/supplementary material, further inquiries can be directed to the corresponding author/s.

## Ethics Statement

The studies involving human participants were reviewed and approved by University of Murcia. The patients/participants provided their written informed consent to participate in this study.

## Author Contributions

AS-d-P carried out the treatment protocol, collaborated on its application as clinical supervisor, corrected the evidence included in the research, and is a collaborator in the writing of the article. LL-C performed statistical treatment and collaborated in the review of articles. MA-L collaborated in the bibliographic search and writing of the introduction, results, and bibliographical references of the article. AM-P, MC-S, and VF-F carried out part of the search for articles, writing of results, and preparation of tables. CL-S designed the research and collaborated in the proofreading and writing of the article. All authors contributed to the article and approved the submitted version.

## Funding

This research was carried out with the support of the Solidarity and Reinsertion Foundation (Murcia) and research funds from the UM.

## Conflict of Interest

The authors declare that the research was conducted in the absence of any commercial or financial relationships that could be construed as a potential conflict of interest.

## Publisher's Note

All claims expressed in this article are solely those of the authors and do not necessarily represent those of their affiliated organizations, or those of the publisher, the editors and the reviewers. Any product that may be evaluated in this article, or claim that may be made by its manufacturer, is not guaranteed or endorsed by the publisher.
